# Co-inhibitor expression on tumor infiltrating and splenic lymphocytes after dual checkpoint inhibition in a microsatellite stable model of colorectal cancer

**DOI:** 10.1038/s41598-021-85810-5

**Published:** 2021-03-26

**Authors:** Ryan J. Slovak, Hong-Jai Park, William M. Kamp, Johannes M. Ludwig, Insoo Kang, Hyun S. Kim

**Affiliations:** 1 Section of Interventional Radiology, Department of Radiology and Biomedical Imaging, Yale School of Medicine, 330 Cedar Street, New Haven, CT 06510 USA; 2 University of Connecticut School of Medicine, 263 Farmington Avenue, Farmington, CT 06032 USA; 3 Section of Rheumatology, Allergy and ImmunologyDepartment of Internal Medicine, Yale University School of Medicine, 300 Cedar Street, New Haven, CT 06520 USA; 4 Warren Alpert Medical School of Brown University, 222 Richmond St, Providence, RI 02903 USA; 5 Department of Diagnostic and Interventional Radiology and Neuroradiology, University Hospital Essen, University of Duisburg-Essen, Hufelandstr. 55, 45147 Essen, Germany; 6 Section of Medical Oncology, Department of Internal Medicine, Yale School of Medicine, 330 Cedar Street, New Haven, CT 06510 USA; 7 Yale Cancer Center, Yale School of Medicine, 330 Cedar Street, New Haven, CT 06510 USA; 8 University of Maryland School of Medicine, 22 South Greene Street, Suite G2K14, Baltimore, MD 21201 USA

**Keywords:** Cancer, Oncology

## Abstract

Checkpoint inhibitors have demonstrated clinical impact in colorectal cancer with deficient mismatch repair and high microsatellite instability. However, the majority of patients have disease with stable microsatellites that responds poorly to immunotherapies. Combinations of checkpoint inhibitors are under investigation as a way of increasing immunogenicity and promoting a robust anti-tumor immune response. The purpose of this study is to quantify the immune responses induced by mono and dual checkpoint inhibition in a mismatch repair proficient model of colorectal cancer (CRC). Tumor growth rates were monitored over time and compared between groups. We utilized fluorescence-activated cell sorting to analyze CD8^+^ and CD4^+^ T cells after treatment with either single PD-1 inhibition or dual PD-1 and CTLA-4 inhibition. Additionally, we sought to quantify the expression of co-inhibitory surface molecules PD-1, LAG3, and TIM3. Dual checkpoint inhibition was associated with a significantly slower growth rate as compared to either mono PD-1 inhibition or control (*p* < 0.05). Neither monotherapy nor dual checkpoint inhibition significantly affected the tumoral infiltration of lymphocytes. After treatment with dual inhibitors, infiltrating CD8^+^ T cells demonstrated significantly less expression of PD-1 (1700 vs. 2545 and 2462; *p* < 0.05) and LAG3 (446.2 vs. 694.4 and 707; *p* < 0.05) along with significantly more expression of TIM3 (12,611 vs. 2961 and 4259; *p* < 0.05) versus the control and anti-PD-1 groups. These results suggest that dual therapy with anti-CTLA-4 and anti-PD-1 antibodies significantly inhibits growth of microsatellite stable CRC by suppressing immunosuppressive checkpoints. Upregulation of TIM3 represents a potential escape mechanism and a target for future combination immunotherapies in CRC.

## Introduction

Immune modulating therapies have demonstrated tremendous efficacy for malignancies such as melanoma and non-small cell lung cancer^1,2^. On the other hand, colorectal cancer (CRC) seems to respond differently based on whether or not the tumor has deficient DNA mismatch repair mechanisms. Colorectal tumors with deficient mismatch repair have high microsatellite instability and high mutational burdens that promote a robust response to treatment with immune checkpoint inhibitors due to an increased generation of neoantigens^3,4^. Unfortunately, no more than 15–23% of CRCs have high microsatellite instability^5^. The majority of tumors are proficient in mismatch repair and exhibit microsatellite stability; as a result, most CRCs tend to respond poorly to immunotherapy^6^.

The immunotherapies that have shown the most promise in managing CRC are immune checkpoint inhibitors. Cancers utilize immune checkpoint pathways to suppress the activation of an anti-cancer immune response^7^. These co-inhibitory pathways include programmed cell death protein 1 (PD-1), cytotoxic T-lymphocyte associated protein 4 (CTLA-4), T cell immunoglobulin mucin-3 (TIM3), and lymphocyte-activation gene 3 (LAG3). Activation of these molecules by ligands expressed on tumor cells contributes to the induction of anergy and exhaustion in cytotoxic T cells^8–10^. Numerous immunotherapeutics have been developed to target and inhibit these pathways. Chief among these are the anti-PD-1 antibodies, which have demonstrated significant efficacy in patients with high microsatellite instability^11^. Many combination immunotherapies are being investigated to further improve clinical response rates^11^. The combination of anti-PD-1 antibodies plus anti-CTLA-4 antibodies in particular has demonstrated significant clinical benefit, however data in microsatellite stable patients remains very limited^12,13^.

With this study, we aimed to elucidate the quantitative differences in immune response induced by single axis PD-1 inhibition versus dual PD-1 and CTLA-4 inhibition. In particular, we were interested in the effect that these treatments had on the majority of CRC patients with microsatellite stability. For this reason, we chose our model as CT26, a line of murine colorectal cancer that does not exhibit mutations in the mismatch repair genes that are associated with high microsatellite instability^14^.

## Materials and methods

### Mice

The Yale Institutional Animal Care and Use Committee (IACUC) approved all procedures performed in this study and all the procedures adhered to the guidelines outlined in the National Institutes of Health Guide for the Care and Use of Laboratory animals and the study was carried out in compliance with ARRIVE guidelines^15^. Thirty BALB/c mice of mixed sex, aged 5–10 weeks and weighing 15–25 g were obtained (Charles River Laboratories, Wilmington, MA, USA).

### Tumor implantation

The CT26.WT (ATCC CRL-2638) murine colorectal cell line was used as our tumor model. To induce tumors, preparations of 0.5 × 10^6^ CT26 cells were subcutaneously injected into both flanks of each mouse. Following bilateral implantation, the tumor cells were given one week to grow inside the mice before treatment began. During this time, the mice were regularly monitored and tumor growth measurements were taken at regular intervals.

### Treatment

After sufficient tumor growth, the mice were randomly assigned to one of three primary treatment groups for a total of 10 mice per group: no treatment (Group I), monotherapy with anti-PD-1 antibodies (Group 2), or dual immune checkpoint blockade (DICB) with anti-PD-1 and anti-CTLA-4 antibodies (Group 3). Each of these main groups was further subdivided into 7 day sacrifices and 14 day sacrifices with 5 mice in each subgroup. Mice in group I received injections of sham IgG antibodies (InVivoMAb IgG controls; BioXcell, West Lebanon, NH, USA) and were used as a control.

Systemic administration of checkpoint inhibitors was performed at three separate time points using intraperitoneal injections of either 200 μg anti-PD-1 (Clone J43; BioXcell) for monotherapy or 200 μg anti-PD-1 plus 100 μg of anti-CTLA-4 (Clone 9D9; BioXcell) for dual therapy. The first injection of checkpoint inhibitors was at the conclusion of the tumor growth period and began “treatment day 0.” The second and third injections of checkpoint inhibitors were performed on treatment day 3 and 5 respectively. Following treatment, average tumor volume (L×W^2^) in both flanks was assessed every other day via caliper measurement. Planned sacrifices were made at 7 and 14 days after treatment. After sacrifice, both tumors and the spleen were also harvested.

### Immune profiling

Immune profiling was performed on the tissue samples taken from and the day 14 sacrifices. Single cell suspensions were made from portions of both tumors and the spleen. These cells were then stained with antibodies for CD45 (BUV395, BD Biosciences, San Jose, CA), CD3 (APC-Cy7, BD Biosciences), CD8 (Pacific Blue, Biolegend, San Diego, CA), CD4 (Alexa 700, Biolegend), PD-1 (FITC, Biolegend), PD-L1 (PE-Cy7, Biolegend), LAG-3 (PE, Biolegend), TIM-3 (APC, Biolegend), and Ki-67 (BV605, Biolegend) or isotype control antibodies before undergoing flow cytometry analysis. All samples were included in a single flow cytometry experiment.

### Statistics

Statistical analysis via non-parametric Mann–Whitney U tests was performed using Graphpad Prism 7 software and statistical significance was defined as *p* < 0.05. Additional scatter plots were made using FlowJo version 10.7 software.

### Ethics approval

The Yale Institutional Animal Care and Use Committee approved all procedures performed in this study.

### Consent for publication

All authors consent for publication.

## Results

### Tumor growth

**Figure 1:**
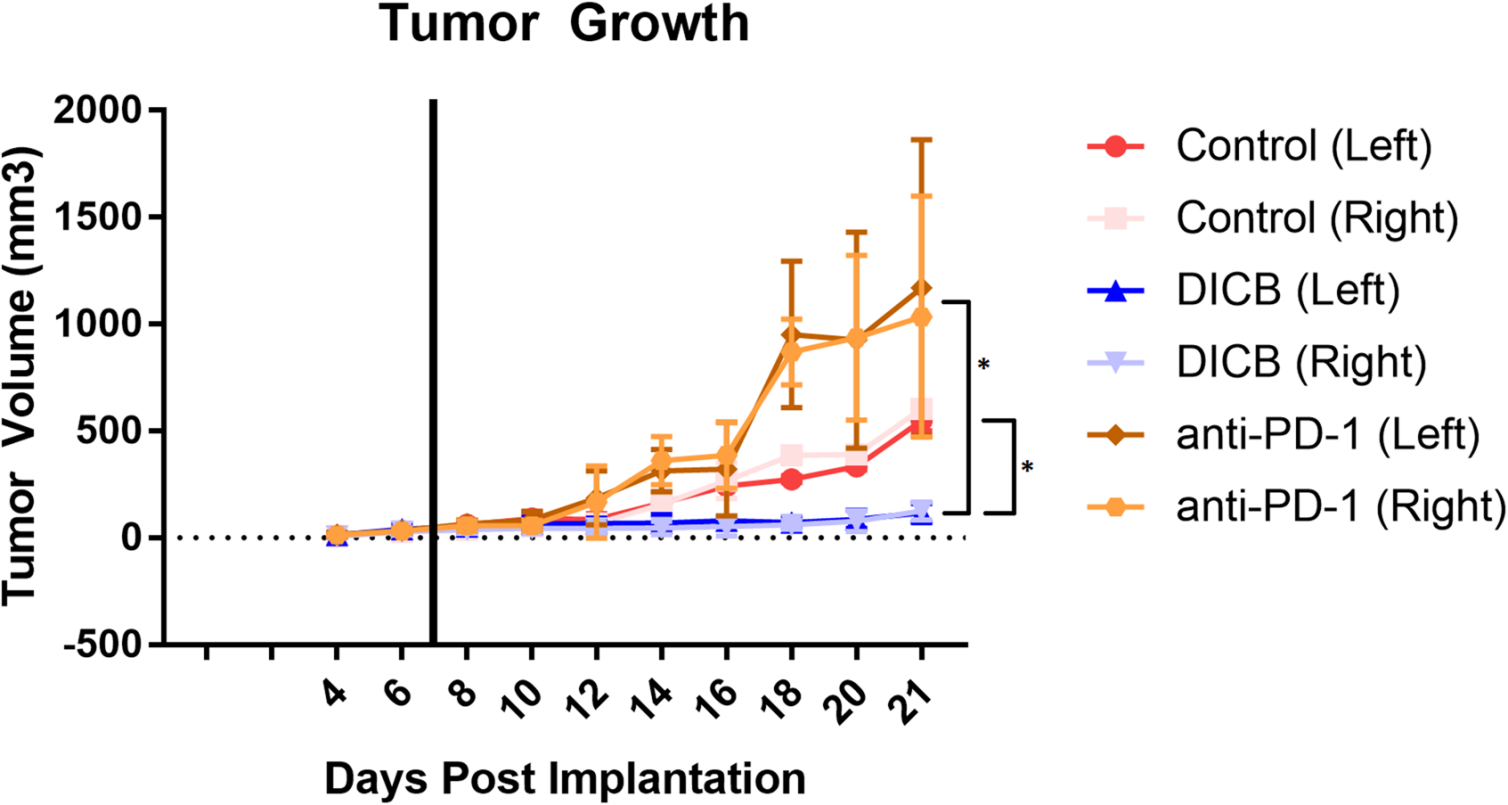
Tumor growth curves for each treatment group, further divided into left sided and right sided tumor growth. The solid black line denotes initiation of treatment. Standard deviation is shown with error bars. Significance of *p* < 0.05 between treatment groups is denoted with an asterisk.

After initiation of treatment, tumor growth in the DICB group was significantly slower than in either the control or the anti-PD-1 group (*p* < 0.05). In spite of this, tumor growth did not stop or reverse in the DICB group. While the tumors in the anti-PD-1 monotherapy group appeared to demonstrate substantially more growth than even the control group, this difference was not statistically significant (*p* > 0.05) (Fig. 1).

### Flow cytometry

**Figure 2:**
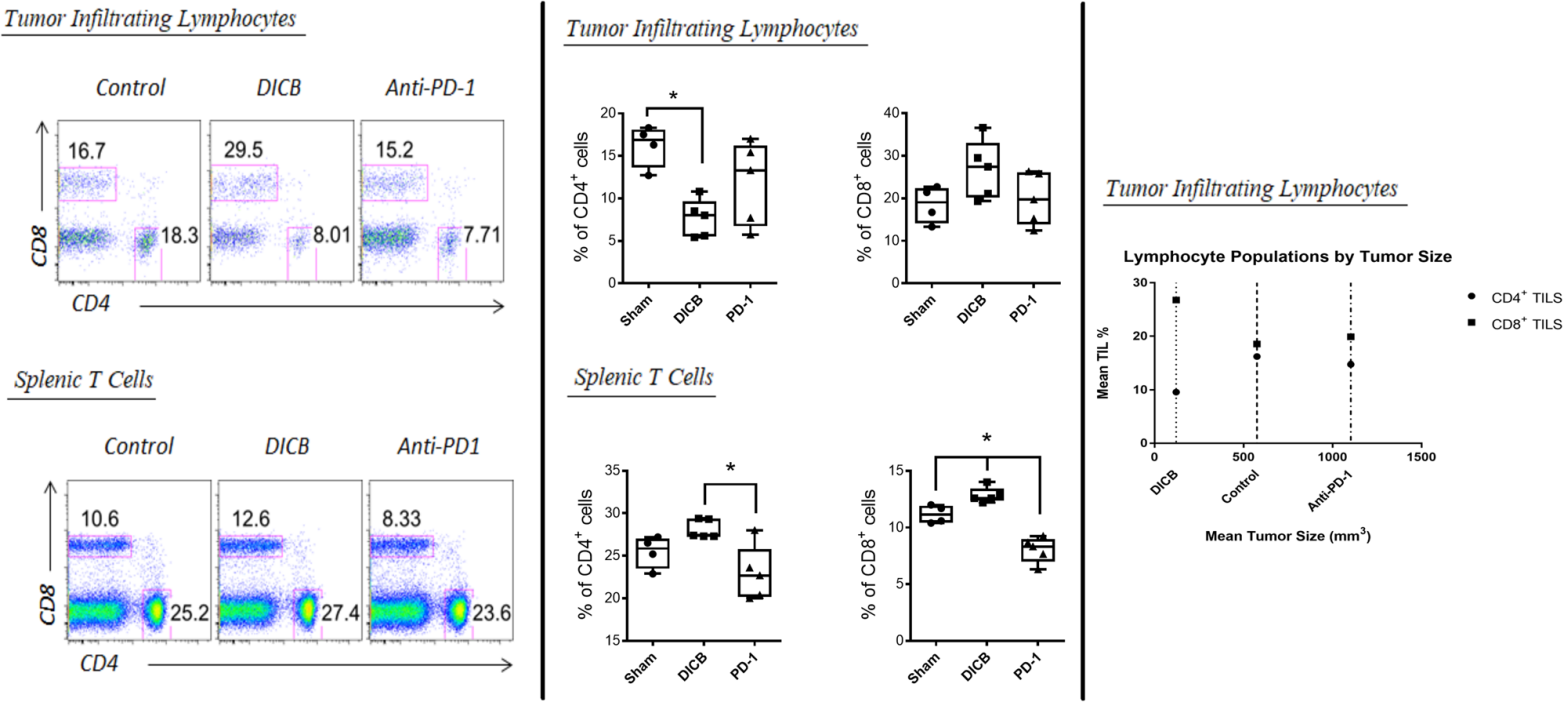
Flow cytometry analysis of tumor infiltrating and splenic CD4^+^ and CD8^+^ T cells divided into 3 panels. The first panel contains scatter graphs depicting the populations of CD4^+^ and CD8^+^ TILs/Splenic T cells for each trteatment group. The second panel shows box and whisker plots illustrating this same data with annotations of significance and the final panel shows the mean percentage of CD4^+^ versus CD8^+^ TILs by tumor size. Significance of *p* < 0.05 is denoted with an asterisk.

Flow cytometry performed on tumor samples from mice sacrificed at day 14 revealed that the percentage of tumor-infiltrating CD8^+^ T cells in animals treated with dual axis inhibition trended higher than the percentage in either the control or the mono anti-PD-1 group, but this difference did not reach statistical significance (27.4 vs. 19.05 and 19.70; *p* > 0.05). Similarly, CD4^+^ T cells appeared to trend downwards in the DICB group when compared to the anti-PD-1 group (8.01 vs. 13.3; *p* > 0.05), and the CD4^+^ T cells were significantly lower in the DICB group versus the control (8.01 vs. 16.90; *p* < 0.05). Among splenic lymphocytes, the patterns observed were notably different. The analysis demonstrated that the number of splenic CD8^+^ T cells was significantly less in the single axis anti-PD-1 group compared to both the control and the DICB groups (8.33 vs. 11.15 and 12.60; *p* < 0.05). Likewise, there were significantly less splenic CD4^+^ T cells in the anti-PD-1 group as compared to DICB (22.70 vs. 27.4; *p* < 0.05), but there was no significant difference between the anti-PD-1 group and the control (22.70 vs. 25.85; *p* > 0.05) (Fig. 2).

### Co-inhibitory molecule expression on TILs

**Figure 3:**
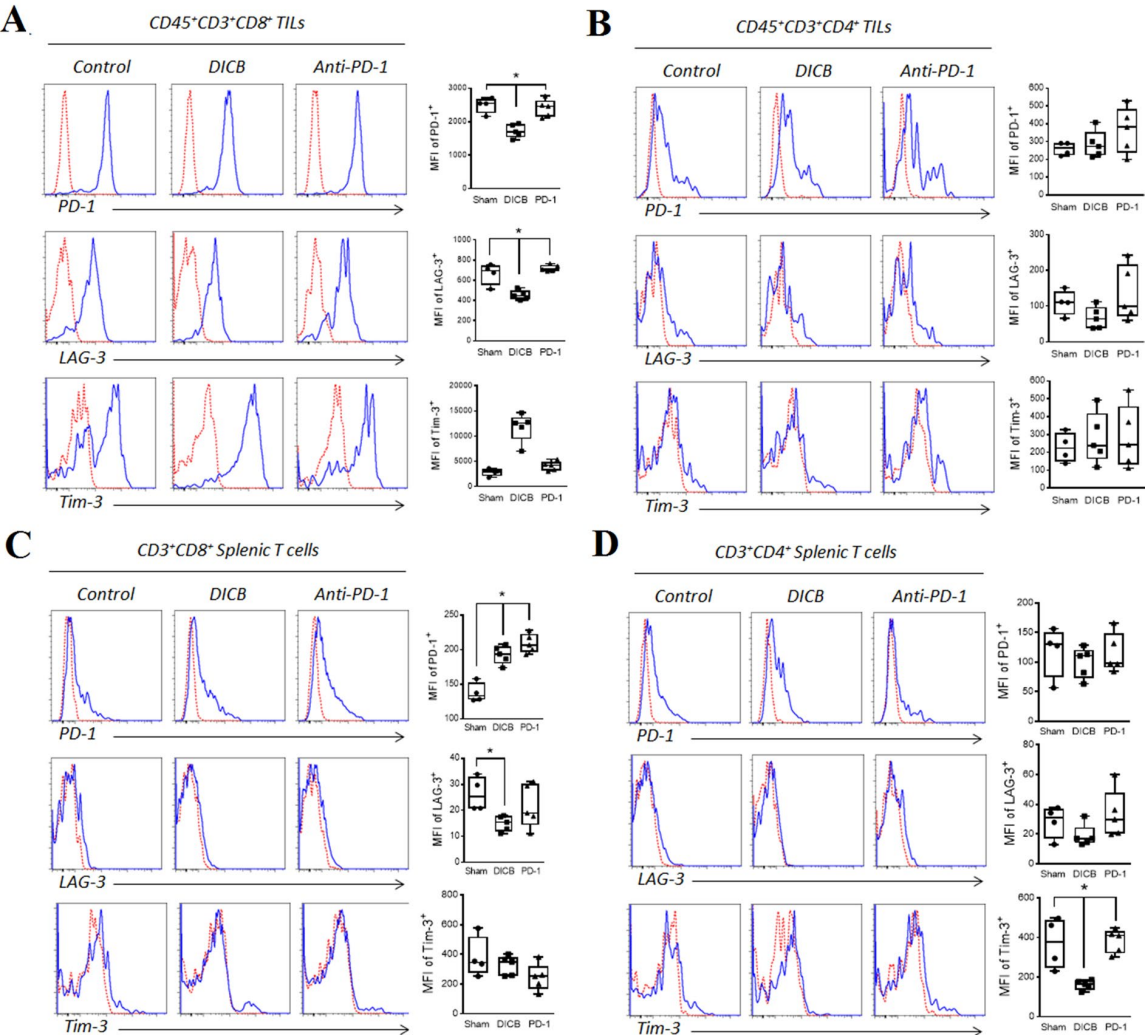
Flow cytometric analysis of co-inhibitory receptors on tumor infiltrating CD8^+^ T cells (**A**), tumor infiltrating CD4^+^ T cells (**B**), splenic CD8^+^ T cells (**C**), and splenic CD4^+^ T cells (**D**) shown as histograms of mean fluorescence intensity (MFI) with matching box and whisker plots. Significance of *p* < 0.05 is denoted with an asterisk.

When examining the expression levels of co-inhibitory receptors on CD45^+^CD3^+^CD8^+^ TILs as measured by mean fluorescent intensity (MFI), it was found that both PD-1 (MFI, 1700 vs. 2545 and 2462; *p* < 0.05) and LAG3 (446.2 vs. 694.4 and 707; *p* < 0.05) were significantly lower in the DICB group as compared to both the control and anti-PD-1 groups. Interestingly, the opposite was true of the checkpoint receptor TIM3, which was significantly higher in the DICB group as compared to the control and the anti-PD-1 groups (12,611 vs. 2961 and 4259; *p* < 0.05) (Fig. 3A). In contrast, no distinct variations between groups were observed in co-inhibitory receptors on CD45^+^CD3^+^CD4^+^ T cells (Fig. 3B).

### Co-inhibitory molecule expression on splenic T cells

The trends in checkpoint receptors observed on splenic T cells were notably different than what was seen on TILs. When CD3^+^CD8^+^ splenic T cells were examined, expression of PD-1 was actually significantly higher in both the DICB and anti-PD-1 groups as compared to control (132.9 vs. 193.7 and 206.8; *p* < 0.05). However, LAG3 expression was significantly lower in the DICB group versus the control (15.48 vs. 25.28; *p* < 0.05) and there was only minimal variation in TIM3 (Fig. 3C). On CD3^+^CD4^+^ splenic T cells, there was little variation in PD-1 and LAG3 expression, but the expression of TIM3 was significantly lower in the DICB group as compared to both the control (164.0 vs. 378.5; *p* < 0.05) and the anti-PD-1 groups (164 vs. 410; *p* < 0.05)(Fig. 3D).

## Discussion

Colorectal cancer is the 3rd most commonly diagnosed cancer and the 2nd leading cause of cancer related deaths in the United States^16^. Further, 25% of all CRC patients will present with metastatic disease at the time of diagnosis and up to 50% of CRC patients will die from metastatic disease^17^. Immunotherapies including PD-1 axis inhibitors in particular have shown some promise in improving outcomes in patients with deficient mismatch repair and high microsatellite instability, but unfortunately this group makes up a small minority of CRC patients. The majority of CRC is proficient in mismatch repair and has not yet proven to be amenable to immunotherapy. For this reason, we aimed to elucidate the specific immune responses elicited by single versus dual checkpoint inhibition in mismatch repair proficient model of CRC.

Similar to several clinical trials that have investigated immunotherapy for microsatellite stable CRC^18,19^, this study demonstrated that monotherapy with PD-1 inhibition had no beneficial impact on the growth of CT26 tumors. This differs slightly from other basic research studies, which have shown that although monotherapy with PD-1 antibodies may not completely eradicate CT26 tumors, it may still result in retardation of tumor growth^20^. Interestingly, the tumors in our study that were treated exclusively with anti-PD-1 antibodies seemed to grow at a more rapid rate than even the control tumors, though this difference did not reach significance due to the large standard deviation in the PD-1 group. Hyperproliferation after single agent PD-1 inhibition has previously been reported in several different cancers^21,22^, including colorectal^23^. It has been theorized that this paradoxical response may be related to a blockade of intrinsic PD-1 expression on tumor cells^24^ or Treg cells^25^, thereby either releasing an intrinsic, anti-survival mechanism or expanding the proliferation of immunosuppressive cells. These significant variations in response to monotherapy with anti-PD-1 antibodies may be due in part to differing levels of intrinsic PD-1 expression and inherent resistance to PD-1 blockade at the time of treatment^24^.

On the other hand, the addition of a CTLA-4 inhibitor significantly slowed growth compared to both the control and the PD-1 groups, although no regression in size was seen. The additional benefit seen with this combination is likely due to the distinct, yet complementary functions of these two molecules. PD-1 limits effector T cell function, whereas CTLA-4 limits T cell activation and expansion^26^. Inhibition of both of these molecules is therefore likely to function synergistically in enhancing the anti-tumor immune response.

The upward trend of tumor infiltrating CD8^+^ T cells seems to suggest that dual checkpoint inhibition promotes a more favorable immune environment. Patients with increased amounts of tumor infiltrating CD8^+^ cells have been shown to have longer overall survival and disease free survival^27–29^ and it is believed that high levels of tumor infiltrating T cells may be a significant contributor to the more robust response to immunotherapy seen in tumors with deficient mismatch repair^4^. The prognostic significance of increased amounts of intratumoral CD8^+^ T cells is perhaps related to the heightened proliferation and cytotoxic activity of these cells, which have previously been demonstrated to express significantly higher levels of Granzyme B and Ki67 than CD8^+^ T cells in nearby stroma^30^. While some studies have suggested that higher levels of tumor infiltrating CD4^+^ T cells may also promote improved clinical outcomes^31^, the various different subsets of CD4^+^ T cells make it difficult to quantify their exact impact^29^. For instance, while Th1 cells may promote anti-tumor immunity by releasing cytokines that activate cytotoxic cells^32^, Th2 and Treg cells have been found to suppress the anti-tumor immune response^33,34^. For this reason, the prognostic value of the decreased tumor infiltrating CD4^+^ T cells observed after DICB is unclear, even though it reached significance when compared to control.

Assessment of splenic T cells has been used to quantify the systemic immune response^35,36^. The finding of significantly less CD8^+^ and CD4^+^ T cells in the spleens explanted from mice in the PD-1 monotherapy group supports the conclusion that PD-1 monotherapy was not beneficial for the anti-cancer immune response, and may in fact have been detrimental. Similarly, though there was a slight upward trend in splenic CD8^+^ and CD4^+^ T cells after DICB, the absence of any major differences suggests that this combination was not sufficient to evoke a robust systemic immune response.

The significant decrease in PD-1 expression on tumor infiltrating CD8^+^ T cells after DICB suggests that this treatment is effectively overcoming the T cell exhaustion induced by this immunosuppressive checkpoint^37^. Similarly, the decrease in LAG3 suggests that this checkpoint is not a limiting factor for the therapeutic efficacy of this treatment; however, the significant increase in TIM3 supports the possibility that upregulation of it or similar, alternative checkpoints may be responsible for maintaining an immunosuppressive environment and dampening the anti-cancer immune response after combined inhibition of PD-1 and CTLA-4. The relevance of these alternative checkpoints has previously been demonstrated in lung cancer patients where the addition of anti-TIM3 antibodies following failure of a PD-1 blockade resulted in a survival advantage^38^.

Notably, the evaluation of co-inhibitory molecules on splenic lymphocytes revealed that the expression of PD-1 was significantly higher after DICB. This suggests that while the treatment may have been sufficient for overcoming the PD-1 checkpoint in the local tumoral environment, it may not have been able to entirely suppress the systemic activity of PD-1.

Taken together, this data suggests that the combination of dual PD-1 and CTLA-4 checkpoint inhibition works to inhibit the growth of microsatellite stable murine model of CRC by promoting cytotoxic T cell infiltration and minimizing the impact of the co-inhibitory molecules PD-1 and LAG3. The presence of additional co-inhibitors such as TIM3 likely acts to limit the efficacy of this treatment. Future investigations should elaborate upon the role that TIM3 and other co-inhibitory molecules have in promoting resistance to dual checkpoint inhibition with anti-PD-1 and CTL-4 antibodies.

## Data Availability

All data and materials are available upon request.
